# Current experiences of accessing and using HIV pre-exposure prophylaxis (PrEP) in the United Kingdom: a cross-sectional online survey, May to July 2019

**DOI:** 10.2807/1560-7917.ES.2019.24.48.1900693

**Published:** 2019-11-28

**Authors:** Charlotte O’Halloran, Greg Owen, Sara Croxford, Lee B Sims, O Noel Gill, Will Nutland, Valerie Delpech

**Affiliations:** 1 National Infection Service, Public Health England, London, United Kingdom; 2 iWantPrEPNow, London, United Kingdom; 3 School of Public Health, Imperial College London, London, United Kingdom; 4 These authors contributed equally; 5 PrEPster, London, United Kingdom

**Keywords:** PrEP, HIV, United Kingdom, men who have sex with men, MSM, sexually transmitted infections

## Abstract

The 2019 online pre-exposure prophylaxis (PrEP) user survey in the United Kingdom was conducted to assess HIV PrEP access, and user characteristics. One in five respondents continued experiencing difficulties accessing PrEP; users were almost exclusively gay or bisexual men at high risk of HIV. The majority obtained PrEP through health service clinics and rated PrEP positively. High STI rates were reported among users. Renal and sexual health checks are advised for those sourcing PrEP privately.

Pre-exposure prophylaxis (PrEP) has been available in the United Kingdom (UK) through online purchase since Autumn 2015, National Health Service (NHS)-funded programmes via sexual health clinics in Scotland and Wales since July 2017, a health service funded trial in England since October 2017 and a risk reduction clinic in Northern Ireland since July 2018. We conducted an online survey to assess PrEP access in the UK, to capture experiences of people living in the UK who are using or report being unable to obtain PrEP since January 2017, and to estimate an upper limit of the number of current users in the UK.

## Survey population

For this survey, a current PrEP user was defined as someone reporting to have taken their first PrEP tablet in July 2019 or earlier and their last PrEP tablet in January 2019 or later.

From 17 May to 1 July 2019, 2,389 participants recruited through the iWantPrEPNow mailing list, social media and Grindr completed the survey. Compared with previous survey years [1], the addition of recruitment through Grindr was new for 2019 (26%; n = 627); sampling changes could have affected observed behaviour differences compared with previous years. Those accessing the survey through Grindr had similar demographics to the total survey sample, except that fewer were living in England (88%; 554/627) and London (32%; 202/627). Participants were eligible for the survey if they were living in the UK and had tried to access or used PrEP since January 2017. Almost all (94%; n = 2,242) identified as exclusively gay and/or bisexual men and the majority (85%; n = 2,041) reported white ethnicity (Table 1). Half of respondents were aged 25–39 years (50%; n = 1,202). The majority (94%; n = 2,241) were living in England, while 3% (n = 73) were living in Scotland, 2% (n = 41) in Wales and 1% (n = 24) in Northern Ireland. Of participants, 78% (n = 1,856) had used PrEP since January 2017 and 94% (n = 1,742) of these were current PrEP users. Of respondents, 114 reported using PrEP since January 2017, but were not current users.

**Table 1:** PrEP user survey: Demographics, sexual behaviour and experiences among current PrEP users and those unable to obtain PrEP, United Kingdom, 2019 (n = 2,275)

Characteristic		Current PrEP user^a^ (n = 1,742)		Tried but unable to obtain PrEP since 2017 (n = 533)	
		n	%	n	%
**Demographics**					
**Gender identity**	Man (including trans man)	1,716	98.5	517	97.0
	Non-binary/In another way	20	1.1	10	1.9
	Woman (including trans woman)	3	0.2	3	0.6
	Not reported	*3*	*0.2*	*3*	*0.6*
**Sexual orientation^b^**	Gay man	1,622	93.1	445	83.5
	Bisexual	110	6.3	75	14.1
	Queer	70	4.0	27	5.1
	Heterosexual	8	0.5	8	1.5
	Gay woman	0	0.0	1	0.2
	Not reported	23	1.3	10	1.9
**Ethnicity**	White	1,488	85.4	460	86.3
	Asian	106	6.1	28	5.3
	Mixed	50	2.9	12	2.3
	Black	45	2.6	17	3.2
	Other	38	2.2	11	2.1
	Not reported	15	0.9	5	0.9
**Age group (years)**	< 20–29	318	18.3	161	30.2
	30–39	650	37.3	161	30.2
	40–49	455	26.1	115	21.6
	≥ 50	315	18.1	95	17.8
	Not reported	4	0.2	1	0.2
**How respondent heard about survey^c^**	Through mailing list/email	917	52.6	195	36.6
	Other (including through Grindr)	450	25.8	240	45.0
	Social media (Twitter, Facebook, Instagram)	312	17.9	88	16.5
	Through a friend	46	2.6	4	0.8
	Not reported	17	1	6	1.1
**Sexual behaviours**					
**Condomless anal/vaginal sex in the past 6 months**	Yes	1,670	95.9	435	81.6
	No	68	3.9	94	17.6
	Not reported	4	0.2	4	0.8
**Number of condomless anal/vaginal sex partners in the past 6 months** (n = 1,670 for current PrEP users and n = 435 for those unable to obtain PrEP)	One	137	8.2	99	22.8
	Two to four	510	30.5	195	44.8
	Five to ten	437	26.2	78	17.9
	More than ten	571	34.2	55	12.6
	Not reported	15	0.9	8	1.8
**Number of condomless anal/vaginal sex partners in the past 6 months on HIV treatment or PrEP** (n = 1,670 for current PrEP users and n = 435 for those unable to obtain PrEP)	I don't know	643	38.5	123	28.3
	None	101	6.0	93	21.4
	One or more	909	54.4	210	48.3
	Not reported	17	1.0	9	2.1
**Used drugs just before or during sex in the past year**	Yes	1,105	63.4	268	50.3
	Yes (chemsex)^d^	724	41.6	85	15.9
	No	612	35.1	262	49.2
	Not reported	25	1.4	3	0.6
**Experiences**					
**Feel satisfied with sex life**	Agree/strongly agree	1,221	70.1	225	42.2
	Neither agree or disagree	324	18.6	160	30.0
	Disagree/strongly disagree	197	11.3	148	27.8
**Has being on PrEP affected your life**	Yes - PrEP has only had a good effect on my life	1,310	75.2	NA	NA
	Yes - PrEP has had a variable effect on my life	203	11.7	NA	NA
	Yes - PrEP has only had a bad effect on my life	4	0.2	NA	NA
	No - PrEP has not affected my life	185	10.6	NA	NA
	Not reported	40	2.3	NA	NA
**Has felt differently treated while using PrEP**	Yes	300	17.2	NA	NA
	No	1,440	82.7	NA	NA
	Not reported	2	0.1	NA	NA
**From whom has felt treated differently by^b^** (n = 300)	Acquaintances and/or strangers	126	42.0	NA	NA
	Dates	118	39.3	NA	NA
	Healthcare provider	79	26.3	NA	NA
	Partners	76	25.3	NA	NA
	Friends	71	23.7	NA	NA
	Family/other	27	9.0	NA	NA
	Not reported	2	0.7	NA	NA

NA: not applicable; PrEP: Pre-exposure prophylaxis.

^a^ Current PrEP users defined as reporting taking their first PrEP tablet in July 2019 or earlier and their last PrEP tablet in January 2019 or later.

**^b^** For this question, respondents could pick multiple answers.

**^c^** Compared with previous years, 2019 survey participants were recruited from Grindr in addition to other recruitment methods.

^d^ Chemsex was defined as using one or a combination of the following drugs before or during sex: crystal methamphetamine, mephedrone and gamma hydroxybutyrate/gamma-Butyrolactone (GHB/GBL).

## Sourcing, experiences and prevalence of PrEP users

Among current users, 62% (1,081/1,742) last sourced PrEP through a trial or programme, including 55% (949/1,742) through the English Impact trial and the remaining either through another trial or a sexual health clinic in Scotland, Wales or Northern Ireland, while 37% (638/1,742) last sourced privately, either through online purchase, buying privately from a clinic, through a friend/re-seller, using PEP as PrEP or obtaining while travelling abroad (Table 2). A small proportion (16%) reported sharing or selling PrEP, most (61%) having originally sourced it online. Of current PrEP users, 75% took PrEP daily and 25% opted for other dosing regimens. The majority (75%) reported PrEP having only had a positive effect on their life (Box), although 17% reported feeling they had been treated differently while using PrEP, most commonly (42%) by acquaintances and/or strangers.

**Table 2:** PrEP user survey: Sourcing among those unable to obtain PrEP and sourcing, sharing, dosing, sexual health and renal function testing among current PrEP users, United Kingdom, 2019 (n =  2,275)

Characteristic		n	%
**Participants who tried but were unable to obtain PrEP since January 2017 (n = 533)**			
**Tried to source PrEP from^a^**	From a sexual health clinic in England as part of the Impact trial	385	72.2
	Buying from the Internet	141	26.5
	Other (including from a friend, when abroad, using PEP for PrEP, from a person reselling a supply, another trial)	126	23.6
	Sexual health clinic in Scotland, Wales or Northern Ireland	22	4.1
	Buying privately from a clinic	16	3.0
	Not reported	14	2.6
**Current PrEP users^b^ (n = 1,742)**			
**Sourcing, sharing and dosing**			
**Last sourced PrEP from^c^**	From a sexual health clinic in England as part of the Impact trial	949	54.5
	Buying from the Internet	538	30.9
	Sexual health clinic in Scotland, Wales or Northern Ireland	103	5.9
	Buying privately from a clinic	64	3.7
	Other (including from a friend, when abroad, using PEP for PrEP, from a person reselling a supply, another trial)	83	4.8
	Not reported	5	0.3
**Ever shared/sold PrEP intended/bought by yourself**	Yes	279	16.0
	No	1,460	83.8
	Not reported	3	0.2
**How this shared/sold PrEP was originally obtained^a^** (n = 279)	Bought from Internet	170	60.9
	From a sexual health clinic in England as part of the Impact trial	76	27.2
	Other (incl. from a friend, when abroad, using PEP for PrEP, from a person reselling a supply, another trial)	21	7.5
	Sexual health clinic in Scotland, Wales or Northern Ireland	9	3.2
	Bought privately from a clinic	3	1.1
**Dosing strategy last time used PrEP**	Daily	1,303	74.8
	Other dosing regimen (including event-based, intermittently, one-off)	439	25.2
**Sexual health and renal function testing**			
**Number of HIV tests in the last 12 months**	None	7	0.4
	One	107	6.2
	Two	226	13.2
	Three or more	1,375	80.1
	Not reported	27	1.5
**Setting of last HIV test^d^** (n = 1,740)	Sexual health clinic	1,523	87.5
	Other setting (including self-sampling, self-testing service and GP)	211	12.1
	Not reported	6	0.3
**Number of STI tests (excluding HIV) in last 12 months**	None	203	11.7
	One	168	9.6
	Two	208	11.9
	Three or more	1,153	66.2
	Not reported	10	0.6
**STI diagnosis in last 12 months**	Yes	882	50.6
	No	850	48.8
	Not reported	10	0.6
**STI(s) diagnosed in last 12 months^a,^** ^e^ (n = 882)	Gonorrhoea	575	65.2
	Chlamydia	537	60.9
	Other	239	27.1
	Not Reported	2	0.2
**If ever obtained PrEP privately, had a renal function test before starting/while taking PrEP** ^f^	Yes	654	37.5
	No	660	37.9
	Not Reported	428	24.6

GP: general practitioner; NR: not reported; PEP: post-exposure prophylaxis; PrEP: pre-exposure prophylaxis; STI: sexually transmitted infection.

**^a^** For this question, respondents could pick multiple answers.

^b^ Current PrEP users defined as reporting taking their first PrEP tablet in July 2019 or earlier and their last PrEP tablet in January 2019 or later.

^c^ Trial/programme sourcing PrEP users defined as PrEP users who last sourced their PrEP either through the Impact trial in England, another trial, or a sexual health clinic in Scotland, Wales or Northern Ireland. Privately sourcing PrEP users defined as PrEP users who last sourced PrEP either through online purchase, buying privately from a clinic, through a friend/re-seller, using PEP as PrEP or obtaining while travelling abroad.

**^d^** This question was only asked of respondents who reported ever having an HIV test.

^e^ This question was only asked of respondents reporting STI diagnoses in the last 12 months.

^f^ This question was asked of all PrEP users, but among current PrEP users last sourcing privately (n = 638), 325 (50.9%) responded ‘Yes’ to having a renal function test, 271 (42.5%) ‘No’ and 42 (6.5%) were not reported.

Box **Examples of current PrEP users’ comments on the positive effects of PrEP on their lives and how they felt they were treated differently, PrEP user survey, United Kingdom, 2019**

**Table:** 

**Positive effect on life from PrEP comments:**
‘The reduced stress. Even when I use condoms, I felt worried that I could get HIV. It definitely hasn't changed my behaviour, I still have as much or as little sex as I would have before. But this has helped me worry less, and also be more happy in myself knowing that I can take some control.’ ‘Lack of fear around all sexual encounters now. Regardless of how safe I was in the past, I always felt there was a 'chance' I could have contracted HIV. Now that chance is so statistically low it's removed any angst around sex.’ ‘I know that I am protected whatever happens. I don't have to rely totally on condoms not failing and I don't have the weeks of anxiety wondering “what if”.’ ‘Much less anxiety about contracting HIV, more STI screenings.’ ‘The relief from the fear of contracting HIV is enormous. I never thought I could have sex without that lingering fear.’ ‘No impending sense of doom when attending for routine STD checks.’
**Feeling treated differently from using PrEP comments:**
‘A lot of people assume I am always looking for unprotected sex and am promiscuous. This is completely false - I use PrEP as an added layer of protection.’ ‘Expectation of unprotected sex as I am on PrEP. People feel that condoms aren't necessary anymore.’ ‘Either positively, being seen as more health conscious and responsible, or negatively, assuming PrEP-users are more promiscuous.’ ‘I have been slut-shamed for using PrEP on hook-up apps. A doctor thought I had HIV because I was on PrEP.’ ‘Almost every healthcare professional I’ve disclosed to about taking PrEP assume I’m HIV Positive’ ‘I do not feel advocacy from healthcare providers and experience a level of judgement.’

PrEP: Pre-exposure prophylaxis; STD: sexually transmitted disease; STI: sexually transmitted infection.

For each private PrEP user who was a non-regular clinic attendee, defined as reporting to have less than two HIV tests in the last 12 months, regardless of setting, there were two private users who were regular clinic attendees, defined as reporting having two or more HIV tests in the last 12 months at a sexual health clinic, and five trial/programme PrEP users (Figure). Using this 1:2:5 ratio, the upper limit of UK PrEP users is estimated to be one-seventh more than the numbers being monitored in clinics.

**Figure:**
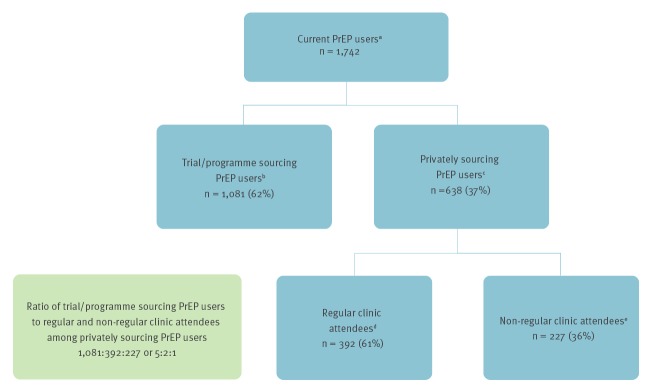
Ratio of current PrEP users sourcing through a trial/programme to those sourcing privately who are regular or non-regular clinic attendees, United Kingdom, 2019 (n = 1,742)

PrEP: pre-exposure prophylaxis.

^a^ Current PrEP users defined as reporting taking their first PrEP tablet in July 2019 or earlier and their last PrEP tablet in January 2019 or later.

^b^ Trial/programme sourcing PrEP users defined as PrEP users who last sourced their PrEP either through the Impact trial in England, another trial, or a sexual health clinic in Scotland, Wales or Northern Ireland.

^c^ Privately sourcing PrEP users defined as PrEP users who last sourced PrEP either through online purchase, buying privately from a clinic, through a friend/re-seller, using PEP as PrEP or obtaining while travelling abroad.

^d^ Regular clinic attendee defined as reporting having two or more HIV tests in the last 12 months at a sexual health clinic.

^e^ Non-regular clinic attendee defined as reporting to have less than two HIV tests in the last 12 months, regardless of setting.

## Sexual behaviour and sexual health

Condomless sex in the past 6 months was reported by 96% of current PrEP users, with a third of these reporting more than ten condomless sex partners (Table 1). Almost a half (45%) reported that either none of these partners were on HIV treatment or PrEP or that they were unsure. In the past year, 63% reported using drugs just before or during sex and 42% reported chemsex.

Of current PrEP users sourcing through trial/programmes 77% (836/1,081), reported three or more STI tests and 92% (994/1,081) three or more HIV tests in the last year. Around a half (56%, 609/1,081) reported an STI diagnosis in the same period, most commonly gonorrhoea (68%, 412/609) and chlamydia (60%; 368/609). Among private users, 48% (308/638) reported three or more STI tests and 58% (367/638) three or more HIV tests in the last year, with 41% (263/638) reporting STI diagnoses.

While all people sourcing PrEP through trial/programmes would have had renal function tests, only 51% (325/638) of current private users reported having one before starting or while taking PrEP, even though two-thirds of private users were regular clinic attendees (Figure).

## Challenges accessing pre-exposure prophylaxis (PrEP)

One in five (22%; 533/2,389) participants reported trying unsuccessfully to obtain PrEP since January 2017. Most (72%) tried to source through the Impact trial and over a quarter (27%) from online retailers (Table 2). Compared with current users, a higher proportion of those unable to access PrEP were aged under 30 years (30% vs 18%), reported bisexual sexual orientation (14% vs 6%) and accessed the survey through other means, including Grindr (45% vs 26%) (Table 1). Rates of condomless sex were also high (82%) among those unable to access PrEP and 13% reported more than ten condomless sex partners. Half reported being unsure or that none of these partners were on HIV treatment or PrEP, and 50% reported using drugs just before or during sex in the past year.

Only 42% of non-users reported feeling satisfied with their sex life, compared with 70% of current PrEP users and smaller proportions of non-users reported chemsex in the past year compared with PrEP current users (16% vs 42%).

## Discussion

Since 2015, steady declines in new HIV diagnoses have been observed among UK GBM [2,3]. This followed the steady scale up of HIV testing over the past decade, the shortening of time to treatment initiation and the recent increase in PrEP use [4-8]. There were around 12,700 people accessing PrEP across England, Wales and Scotland through health service trials and programmes at the end of 2018 [6-8]. From our ratio of users sourcing through a trial/programme and privately (Figure), we estimate up to 7,600 others are sourcing PrEP privately. Of concern, we show that half of individuals sourcing PrEP privately are not undergoing baseline and ongoing renal function assessments, despite the majority being regular clinic attendees.

Our findings confirm that current PrEP users are at high-risk of exposure to HIV; the majority reported condomless sex with multiple partners and sexualised drug use, and many also reported condomless sex with partners of unknown HIV status or HIV treatment or PrEP status. The high STI rates reported in current PrEP users in this survey are consistent with recent reports in other countries [9,10] and may reflect more frequent testing. Services providing PrEP should consider how they can mitigate STI risk through integrating PrEP provision with STI screening and treatment to enhance STI control [11]. A high proportion of current users reported taking daily PrEP despite previous published evidence that event-based oral PrEP is highly effective in preventing HIV [12-14]. PrEP had a strong positive effect on the lives of users and the majority were satisfied with their sex life.

Although PrEP is highly effective for HIV prevention, PrEP availability and scale-up across Europe has been slow and uneven to date, and the ‘gap’ between self-reported use and expressed need is large across European countries [15]. Additionally, our survey highlights high demand for PrEP and a larger PrEP ‘gap’ than expected, with around one in five individuals in need of PrEP but unable or unsure of how to access publicly funded PrEP. Many of them were at high risk of HIV and would have benefited from PrEP and the proportion unable to access PrEP is similar to findings from the PrEP user survey conducted in the previous year [1], despite recent increases in the number of Impact trial places. However, this survey asked about experiences since January 2017 so the finding may be an expression of intermittent capping of trial places and capacity strains at trial clinics [8]. As other European countries are implementing pilot PrEP programmes or localised schemes, this report illustrates the value of estimating the true scale of PrEP need.

Finally, while knowledge of PrEP among UK GBM is among the highest in Europe [16], a small but appreciable number of users reported a knowledge gap among healthcare providers, including negative judgements. Greater awareness among health professionals is required to ensure these experiences do not undermine access or adherence to PrEP.
